# A new eggshell-derived calcium phosphate bioceramic for tissue engineering: cytotoxicity and histomorphometric study

**DOI:** 10.1590/acb402625

**Published:** 2025-03-31

**Authors:** Conrado Dias do Nascimento, Laisa Kindely Ramos de Oliveira, Amy Brian Costa e Silva, Patrícia Roccon Bianchi, André Gustavo de Sousa Galdino, Daniela Nascimento Silva

**Affiliations:** 1 Universidade Federal do Espírito Santo – Programa de Pós-Graduação em Ciências Odontológicas – Vitória (ES) – Brazil.; 2 Universidade Federal do Espírito Santo – Curso de Graduação em Odontologia – Vitória (ES) – Brazil.; 3 Associação Brasileira de Odontologia – Seção Espírito Santo – Vitória (ES) – Brazil.; 4 Instituto Federal de Educação, Ciência e Tecnologia do Espírito Santo – Vitória (ES) – Brazil.

**Keywords:** Biocompatible Materials, Egg Shell, Phosphates, Tissue Engineering

## Abstract

**Purpose:**

To evaluate cytotoxicity and tissue repair of a new chicken eggshell-derived bioceramic (hydroxyapatite/dicalcium phosphate anhydrous–HA/DCPA).

**Methods:**

Cytotoxicity was evaluated in fibroblasts (L cell, L-929) by MTT test. Tissue repair of HA/DCPA was compared to HA/β-TCP bioceramic (Maxresorb-MXR). Two critical-sized bone defects (CSDs) were drilled in the calvarial of 24 Wistar rats and filled with one of the biomaterials. The animals were euthanized after 30, 60, and 90 days, and bone specimens were examined by histomorphometric analyses, scanning electron microscopy, and energy-dispersive X-ray spectroscopy. The percentages of newly formed bone, connective tissue, remaining biomaterial, and total tissue repair area were compared between groups using Student’s t-test and analysis of variance (*p* ≤ 0.05).

**Results:**

HA/DCPA did not exhibit any cytotoxicity. CSDs contained newly formed bone from the defect margins and from ossification centers interspersed throughout the biomaterials. At 30 days, HA/DCPA group had a significantly larger total tissue repair area than MXR group (*p* = 0.047). No differences were observed between groups regarding variables studied (*p* > 0.05).

**Conclusion:**

HA/DCPA is non-cytotoxic. This cement promoted new bone formation and tissue filling of the entire defect area with degree of biomaterial degradation similar to HA/β-TCP, proving to be equally suitable and successful for bone regeneration.

## Introduction

Bioceramics constitute a wide range of calcium phosphate-based, inorganic, non-metallic, biocompatible materials^1^^,^ 2 used in a variety of applications, ranging from cements and metallic implant coatings to filling of cavitary bone defects, due to their biomimetic and osteoconductive properties^3^^–^ 5. Among currently available bioceramics, hydroxyapatite (HA), tricalcium phosphate (TCP), and dicalcium phosphate anhydrous (DCPA or monetite) stand out for their attractive biological properties for tissue engineering^6^^–^ 8.

Zanelato et al.^9^ developed and described a biphasic calcium phosphate (BCP) bone cement composed of HA/DCPA obtained from chicken eggshells. The experimental specimens demonstrated mechanical strength, porosity, and setting time with values greater to those of some calcium phosphate-based cements available on the market.

Eggshell waste has been ranked as the 15th most serious pollution problem in the food industry. Eggshells constitute a valuable raw material, mainly due to the presence of calcium carbonate in their composition; it is a particularly promising form of biowaste^10^ for potential medical and dental applications^11^^,^ 12.

HA/DCPA-based BCPs combine the positive properties of these two biomaterials, yielding a more soluble and stable compound which provides better outcomes in terms of bioactivity, bioresorbability, and osteoconductivity^13^. Obtaining HA/DCPA from chicken eggshells would add further benefits, including low cost and an essentially inexhaustible source of calcium carbonate^9^.

The influence of eggshell on bone regeneration has been investigated in preclinical studies, but no *in-vitro* or *in-vivo* trials of eggshell-derived HA/DCPA were found in the literature^14^. The present study aimed to evaluate the *in-vitro* cytotoxicity and *in-vivo* tissue repair process with a BCP cement composed of HA/DCPA obtained from chicken eggshell.

## Methods

### Biomaterials

- HA/DCPA (84.5% HA + 15.5% DCPA): BCP was produced by the chemical precipitation from the calcination of chicken eggshell at 930°C to convert the raw material into calcium oxide supplied in powder form9. As recommended by the authors15, this compound should be used in the form of a cement from the HA/DCPA powder was compounded with the reaction accelerator disodium hydrogen phosphate decahydrate (Na2HPO4·12 H2O), diluted in 2.5% distilled water at the proportion of 1.1 mL/g, and carboxymethylcellulose (CMC) (Denvercel PH-40A), at the concentration of 3.2% by mass;
- HA/β-TCP (60% HA 40% β-TCP): alloplastic graft is marketed in Brazil under the brand name Maxresorb (MXR, Straumann) and supplied in the form of granules.

### X-ray diffractometry

Samples of the two compounds were examined by X-ray diffraction (XRD-6000, Shimadzu) using Cu Kα radiation with a wavelength of 1.5418 Å, voltage 40 kV, and current 30 mA. The diffraction angles (2θ) of each sample were measured from 20 to 60° at a speed of 2°/min. The X-ray diffractogram was prepared in OriginPro 2019 software (OriginLab).

### In-vitro cytotoxicity testing of HA/DCPA cement

*In-vitro* cytotoxicity testing was performed at the Rio de Janeiro Cell Bank (Polo Tecnológico de Xerém, Instituto Nacional de Metrologia, Qualidade e Tecnologia), following ISO 10993-5 standards^14^. To obtain conditioned media, HA/DCPA samples in powder form (0.2 g/mL) and sodium dodecyl sulfate (SDS) reference material (0.05 mg/mL) were extracted in culture medium for 24 h at 37°C with 5% CO_2_, following ISO 10993-12.

Fibroblasts (NCTC clone L929) were seeded in 96-well microplates at the density of 1.0×104 cells/100 μL and cultivated in Eagle’s minimal essential medium (MEM) supplemented with 10% serum and 1% antibiotic solution (penicillin 100 IU/mL, streptomycin 100 μg/mL).

The 96-well plates were incubated in a humidified oven (37°C, 5% CO_2_) for 24 h to allow sedimentation and cell adhesion. The HA/DCPA and reference group (SDS: positive control) extracts were added to the culture wells; some wells were kept aside as vehicle control group (no extracts added: VCG). After 24 h of exposure, the medium was removed, the wells containing cells were carefully washed with 200 μL of Dulbecco’s phosphate-buffered saline (D-PBS; GIBCO), and 100 μL of MTT dye solution [3-(4,5-dimethylthiazol-2-yl)-2,5-diphenyltetrazolium bromide, 0.5 mg/mL] added to each well. The microplates were incubated at 37°C with 5% CO2 for 2 hours. At the end of the reaction period, the MTT dye was discarded and replaced with 100 μL of acidified isopropanol solution (isopropanol with 0.04 N hydrochloric acid, Sigma). The microplates were incubated for 30 min in the dark at room temperature for solubilization of formazan crystals. The colorimetric assay was performed in triplicate.

For quantitation of formazan, the optical densities (OD) of each well were read on a spectrophotometer at the wavelength of 570 nm. Cell viability, expressed as a percentage (viab%), was calculated according to ISO 10993-5 standard Eq. 1:


$$\mathrm{Viab}(\%)=\frac{\text{OD570a or OD570b}}{\text{OD570c}}\times 100$$ Eq. 1

where: OD570a: the OD of the HA/DCPA; OD570b: the OD of the positive control; OD570c: the vehicle control.

### In-vivo essays

The experiment on animals was approved by the Commission on Ethics in the Use of Animals (number 30/2020) complying guidelines from the National Council for the Control of Animal Experimentation. Twenty-four adult male Wistar rats (body weight 250–300 g), obtained from the Universidade Federal do Espírito Santo vivarium, were used. Briefly, two critical-sized defects (CSDs) were drilled into the calvarial bone of each animal, for a total of 48 defects, allocated across two groups according to biomaterial (HA/DCPA or MXR) and analyzed at 30, 60, and 90 days.

General anesthesia was induced with 10% ketamine (100 mg/kg–0.1 mL/100 g, Ketamine Agener) and 2% xylazine (10 mg/kg–0.05 mL/100 g, Anasedan), intraperitoneally. Enrofloxacin 2.5% (10 mg/kg, Flotril, Intervet Schering-Plough) was administered subcutaneously 1 hour before the surgical procedure. Morphine (5 mg/kg, Dimorf) was administered by subcutaneous injection into the dorsal cervical region. After anesthetic infiltration with 2% lidocaine hydrochloride with 1:100,000 epinephrine (Alphacaine 100, DFL) subcutaneously, it was made a linear coronal incision of 1.5 cm.

CSDs 5 mm in diameter were drilled into the parietal bones, one on the right and one on the left, with a pear-shaped multilaminate drill with an external diameter of 5 mm (i.e., corresponding to the defect size). The biomaterials were then placed into the defects according to group allocation. Defects filled with MXR were covered with a biological membrane (type I collagen of bovine origin, Green Regener EIRELI). The edges sutured with simple interrupted stitches using 5-0 nylon.

After 30, 60, and 90 days, the animals were euthanized with a lethal dose of 10% ketamine (300 mg/kg–0.3 mL/100 g) and 2% xylazine (30 mg/kg–0.15 mL/100 g), intraperitoneally. The calvaria were dissected and removed *en bloc* and stored in 10% buffered formalin for 48 hours for histological processing.

### Histomorphometric analysis

The calvaria (n = 7 per group) containing the CSDs were cut into slices 6-µm thick with a microtome (RM2125 RTS, Leica Biosystems Nussloch GmbH, Nussloch, Germany), stained with hematoxylin and eosin (H.E.), and examined under light microscopy at 4X and 10X magnification. Histomorphometric analysis was performed using ImageJ 1.50i software (Madison, WI, United States of America) by means of delimitation of the perimeter of the area of interest to obtain the following measurements (all as percentages): newly formed bone area, connective tissue area (including fibrous tissue, blood vessels, and adipose tissue), remaining biomaterial, and total tissue repair area. The examiner was calibrated using the intraclass correlation coefficient^16^. The measurements thus obtained were tabulated in a Microsoft Excel spreadsheet (Microsoft, Redmond, WA, United States of America) for further statistical analysis.

### Scanning electron microscopy and energy-dispersive X-ray spectroscopy

Samples (n = 1 per group) were processed and evaluated by scanning electron microscopy (SEM) (JSM-6610LV, JEOL Ltd.) at 15 kV and different magnifications. After SEM, samples were analyzed by energy-dispersive X-ray spectroscopy (EDS-X, Flash Detector 6|10), at 30 kV and a focal length of 25 mm. The different X-ray energy levels were analyzed on proprietary software embedded in the device.

### Statistical analysis

All statistical analyses were carried out in IBM Statistical Package for the Social Sciences Statistics, version 24.0 (IBM Corp.). Student’s *t*-test for independent samples was used to compare the percentages of newly formed bone, remaining biomaterial, connective tissue, and total tissue repair area between the HA/DCPA and MXR groups. Analysis of variance (ANOVA) was performed for within-group assessment of tissue repair over the course of the experimental period. The significance level was set at 5% (*p* < 0.05) for all analyses.

## Results

### Characterization of biomaterials

The HA/DCPA consists of finely distributed solid particles, of spherical morphology, smaller than 30 μm and porosity ~18%^9^. The MXR is composed of rough solid particles of irregular morphology, with a size between 500–1,000 μm and porosity ~80% (Straumann, manufacturer information).

**Figure 1:**
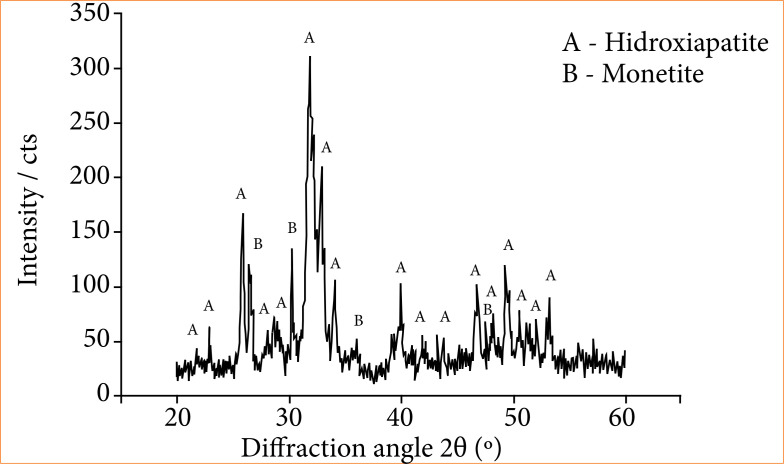
X-ray diffraction analysis of bioceramics: hydroxyapatite/dicalcium phosphate anhydrous (HA/DCPA) hydroxyapatite peaks and monetite peaks.

Source: Elaborated by the authors.

X-ray diffraction analysis of HA/DCPA cement confirmed that the biomaterial consists of a mixture of HA [Ca_4,776_(H_0,39_(PO_4_)_3_(OH)_0,942_] and DCPA [CaPO_3_(OH)] phases (Fig. 1).

### In-vitro cytotoxicity

**Figure 2:**
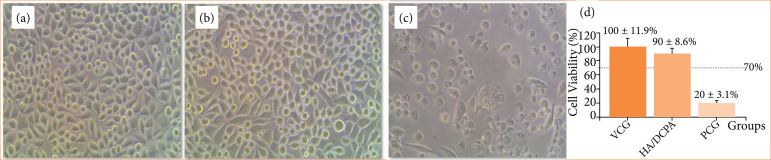
Photomicrographs of the test system (NCTC clone cells [L cell, L- 929, derived from Strain L]). After 24 h of exposure to **(a)** test culture medium (vehicle control group–VCG); **(b)** extract of the hydroxyapatite/dicalcium phosphate anhydrous (HA/DCPA) (24 h extraction time); **(c)** 0.05 mg/mL sodium dodecyl sulfate extract (positive control group–PCG) (200x magnification); **(d)** percentage of cell viability determined by the MTT method.

Source: Elaborated by the authors.

The population and morphology of NCTC clone L929 fibroblasts from the test group were unchanged after 24 h of exposure to the conditioned medium obtained from HA/DCPA extract (24 h extraction time) as compared to the VCG (Figs. 2a and 2b). As expected, the positive control group (PCG) proved cytotoxic, with very low levels of cell survival (Fig. 2c). The cell viability of the biomaterial was determined by comparison the highest optical density measured by the MTT assay in the VCG. After exposure to HA/DCPA granules (powder) for 24 h, cell viability was 90 ± 8.6% as evaluated by the MTT method, while the PCG reduced cell viability to 20 ± 3.1% (Fig. 2d). The cell viability value > 70% demonstrated that the tested HA/DCPA product has no cytotoxic potential.

### Histologic analysis

**Figure 3:**
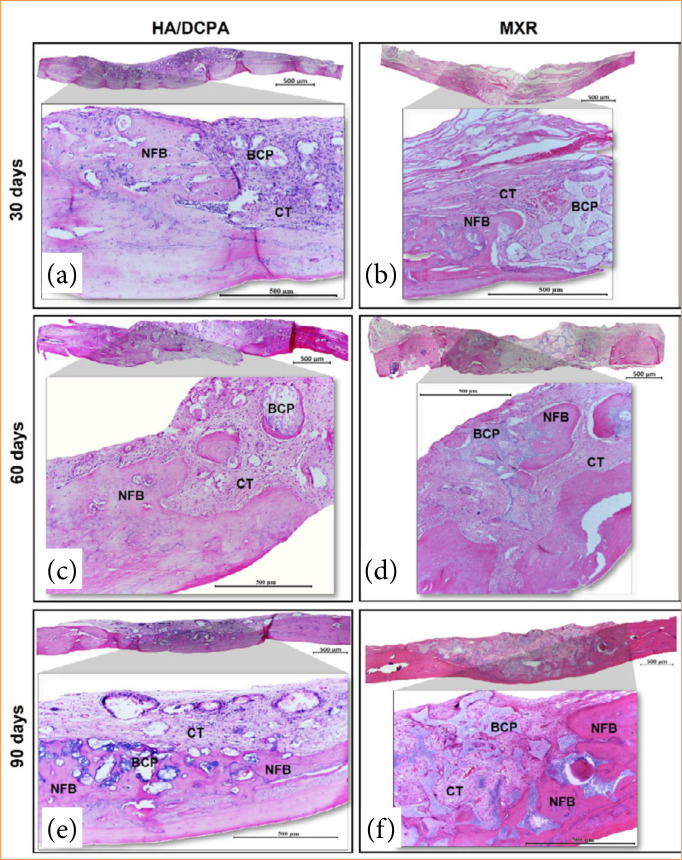
Photomicrograph of the area of tissue repair of bone defects (5 mm). Tissue repair pattern with areas of newly formed bone (NFB), connective tissue (CT) and remaining biomaterial (BCP). The animals were evaluated at (**a, b**) 30, (**c, d**) 60 and (**e, f**) 90 days after the grafting procedures (4X and 10X magnification, hematoxylin and eosin staining).

Histologic examination showed that HA/DCPA and MXR placement resulted in new bone formation *in vivo* at all the three time points of analysis (Fig. 3). The newly formed bone within the bone defects is characterized by the presence of immature bone, with osteoclasts in its lacunae, which distinguishes it from the mature or lamellar bone pre-existing in the calvaria. In both groups, highly cellular loose connective tissue developed in the area adjacent to the defect margin and around the implanted biomaterials. Inward bone growth from the defect margins occurred progressively over time. At 30 days, there was formation of new bone tissue from the margin to the center of the defects filled with HA/DCPA, with visible blood vessels and osteogenic cells both on the surface and within the biomaterial. In the defects filled with MXR, a greater disposition of collagen fibers, more empty spaces, and a smaller volume of biomaterial were observed (Figs. 3a and 3b). At 60 days, the repair process was similar in both groups. At this time point, there was visible bone formation at the center of the defect, no longer contiguous with the defect margins, between and within biomaterial particles. Tissue volume was preserved within the defects, although the volume of newly formed bone appeared to be smaller in the HA/DCPA group than in the MXR group (Figs. 3c and 3d). At 90 days, the degree of repair in both groups was again similar. In the HA/DCPA group, a greater number of osteogenic cells and blood vessels were seen, and the newly synthesized bone matrix showed greater integration with the biomaterial compared to the defects filled with MXR (Figs. 3e and 3f). Some defects filled with MXR showed absence of biomaterial.

HA/DCPA: hydroxyapatite/dicalcium phosphate anhydrous; MXR: Maxresorb. Source: Elaborated by the authors.

### Histomorphometric analysis

**Figure 4:**
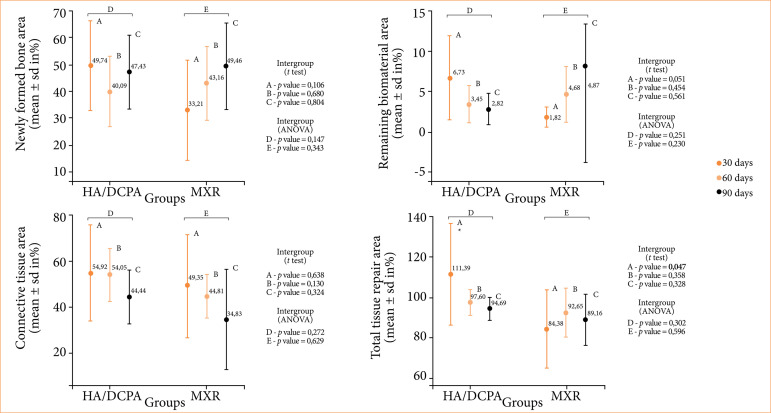
Graphical representation of the histomorphometric analysis (mean ± standard deviation in %; n = 5 group/experimental periods) of bone defects: newly formed bone area, remaining biomaterial, connective tissue, and total repair tissue area. (A, B and C) Student’s t test for intergroup samples; (D and E) ANOVA for intragroup samples.

No differences were observed between the experimental groups regarding percentage of newly formed bone, connective tissue, and remaining biomaterial at the three time points of observation (*p* > 0.05). However, the HA/DCPA group showed a total tissue repair area greater than that of the MXR group at 30 days (*p* = 0.047) (Figs. 3 and 4), with no difference between the two biomaterials at subsequent time points. At 90 days, both biomaterials were similar in terms of new bone formation within- or inter-group comparison (Fig. 4).

HA/DCPA: hydroxyapatite/dicalcium phosphate anhydrous; MXR: Maxresorb; ANOVA: analysis of variance; *significant difference (*p* ≤ 0.05). Source: Elaborated by the authors.

### Scanning electron microscopy and energy-dispersive X-ray spectroscopy

**Figure 5:**
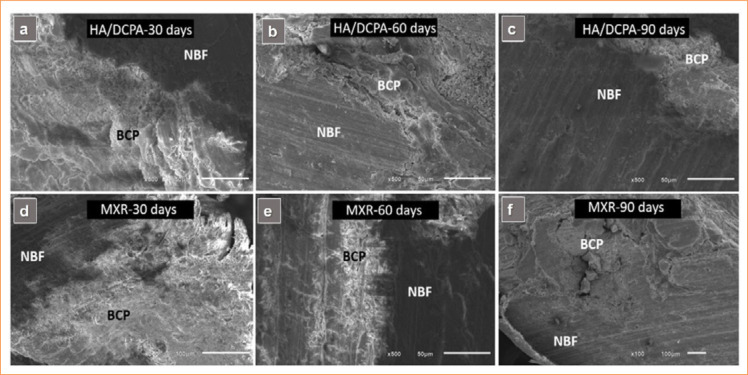
Micrographs of bone defects after implantation of biomaterials. Scanning electron microscopy: HA/DCPA at **(a)** 30, **(b)** 60 and **(c)** 90 days; and MXR at **(d)** 30, **(e)** 60 and **(f)** 90 days, showing newly formed bone (NFB) and remaining biomaterial (BCP). 1,221 × 561 (300 dpi).

**Figure 6:**
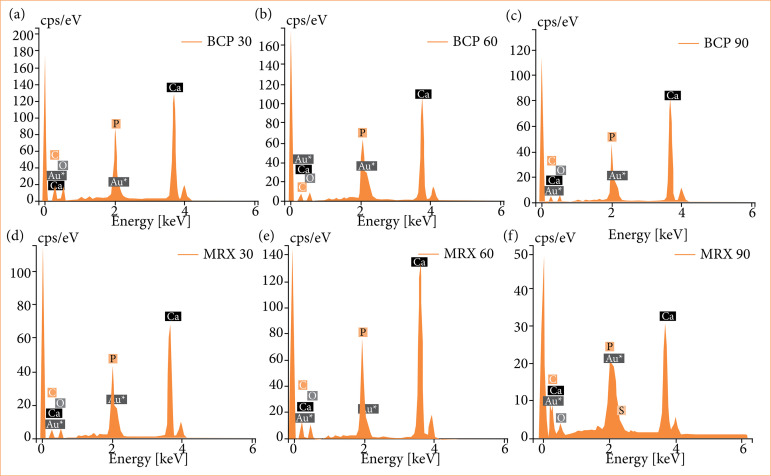
Energy dispersive X-ray spectroscopy (EDS) demonstrates the presence of (a, b, c, d, e and f) oxygen **(O)**, magnesium (Mg), carbon **(C)**, calcium (Ca), phosphorus **(P)** and gold (Au) in all periods in both materials, and **(f)** S (sulfur) at 90 days in the MXR-90. 4,192 × 2,425 (300 dpi).

SEM demonstrated the presence of remaining biomaterial and newly formed bone from the periphery toward the center of the bone defects in both groups. Some more opaque and other more translucent areas were observed. The surfaces of these structures suggest an interface between the newly formed bone and the biomaterial (Fig. 5). At all three time points of observation and in both experimental groups, energy-dispersive X-ray spectroscopy analysis revealed major peaks indicating the presence of oxygen (O), carbon (C), and calcium (Ca) and phosphorus (P) peaks even more intense than those of gold (Au) used in pre-SEM specimen coating, which denotes–in addition to the presence of the biomaterial–one of the stages of the bone mineralization process (Fig. 6).

HA/DCPA: hydroxyapatite/dicalcium phosphate anhydrous; MXR: Maxresorb. Source: Elaborated by the authors.

BCP: remaining biomaterial MXR: Maxresorb. Source: Elaborated by the authors.

## Discussion

As it is a novel product, there are no data in the literature on biological responses to the HA/DCPA cement synthesized by Zanelato et al.^9^. Microstructural characteristics such as size, shape, and distribution of HA/DCPA granules were previously analyzed by the original authors using SEM; mechanical strength, porosity, and setting time were also tested^9^. Thus, our study investigated the *in-vitro* cytotoxicity and *in-vivo* bone regeneration properties of this compound and compared it to MXR, a commercially available product already used in clinical practice. Our motivation for using chicken eggshell was the fact that phosphates obtained from this waste biomaterial have demonstrated excellent properties in the treatment of bone defects *in vivo* in experimental models^8^.

Chicken eggshells are a natural source of calcium phosphates that is economically viable and highly advantageous, as they are usually a waste byproduct from other human activities and can be obtained at low or no cost when compared to synthetic sources of these compounds. Furthermore, certain raw materials used in the synthetic route to obtain calcium phosphates are hazardous and corrosive^11^.

The cytotoxicity of HA/DCPA was evaluated by the MTT test, an easy, accurate, sensitive, specific, fast, low-cost quantitative colorimetric technique^17^ that is a widely accepted method for evaluation of the toxicity of bioceramic materials^18^. HA/DCPA was associated with preservation of cell populations and morphology on MTT testing. In the positive control group, exposure to the SDS extract greatly reduced the number of viable cells compared to the vehicle control group, as expected. This demonstrates that the tested biomaterial lacks cytotoxic potential. *In-vitro* studies have evaluated the cytotoxicity of chicken eggshell-derived calcium phosphates in different cell lines by the MTT method and demonstrated preservation of cell morphology and behavior, as well as greater cell viability, compared to commercially available lyophilized bovine bone graft^12^^–^ 15. Such findings encouraged the performance of *in-vivo* tests, using the Wistar rat calvarial bone defect model for experimental surgery^19^^,^ 20. This model allows easy access to bone tissue and does not affect the animals’ masticatory or locomotor systems, reducing the risk of complications. In addition, because its development and intramembranous ossification are similar to those of the maxilla and mandible, the calvaria is a particularly appropriate site for the evaluation of biomaterials intended to fill intraoral or maxillofacial defects^2^.

The HA/DCPA used in this study is a powder composed of finely dispersed particles smaller than 30 μm in diameter, with spherical morphology and porosity of 18%9. Powders composed of spherical (round) particles with an average size of 2–20 μm are ideal for application in cement form^21^. MXR consists of 60% HA/40% β-TCP and is available as 500–1,000 μm granules, with an approximate porosity of 80% (Straumann Brasil). The HA/β-TCP composite scaffold at a 60:40 ratio of MXR allows a close interface with the adjacent bone and has not been shown to fail in the early stages post-implantation^22^. The size of the granules is an important factor in the repair process, and β-TCP particles with a diameter in the 250–500 μm range provide optimal bone growth^3^.

Porosity is one of the parameters that affects both the biological activity and mechanical properties of a graft material^23^. Even though MXR granules are substantially larger than HA/DCPA particles, its high porosity may explain some of the similarities between the patterns of bone growth observed with these two biomaterials.

HA/DCPA was easy to handle and place into the surgical sites, allowing better fit to the defect cavity and better volume control of the grafted material, with no displacement. These advantages can be attributed to the formulation of the biomaterial. Calcium phosphate cements in paste form are injectable, solidify *in situ*, and are able to take on the shape of complex and even vertical bone cavities; they are also indicated for use in minimally invasive procedures^13^. Recent studies show that injectable MXR has similar results to anorganic bovine bone for the purpose of alveolar ridge augmentation after tooth extraction and dental implant placement^24^^,^ 25. Injectable MXR was not used in this study, because it is not commercially available.

The fact that MXR is only available in granule form makes it more difficult to handle in clinical practice; it must be covered with a biological membrane over the defect site to prevent displacement of the granules even when well adapted to the defect and placed into horizontal cavities.

In the present study, the biological membrane was used only in the MXR group with the aim of keeping the granules in place and preventing their postoperative displacement. In the HA/DCPA group, due to the biomaterial being presented in the form of cement, it does not displace from its position after insertion, thus not requiring the overlay of the biological membrane to maintain the volume of the biomaterial in the bone defect. Studies show that the membrane may interfere with the speed of graft osteointegration and the biodegradation of the biomaterial^26^. It is observed that, even with the presence of the membrane in the MXR group, at the end of the experiment, there was no difference between the groups regarding the percentages of newly formed bone and remaining biomaterial.

One of the limitations of this study is the lack of comparison with other BCP cements of similar composition and configuration since they either do not exist or are not commercially available in Brazil. Future research directions could include implantation of HA/DCPA in load-bearing anatomical regions and comparison to BCPs with the same pharmaceutical form for *in-vivo* evaluation of the mechanical properties of this biomaterial.

The results of the *in-vivo* experiments of this study showed new bone formation for both biomaterials tested, with no osteolytic reaction, further confirming the biocompatibility of HA/DCPA as already established *in vitro*. Neither HA/DCPA nor MXR had been completely resorbed by the end of the study period. However, on histomorphometric analysis, the HA/DCPA group showed a total tissue repair area greater than that of the MXR group at 30 days, with no difference between the two biomaterials at subsequent time points.

## Conclusion

Eggshell-derived HA/DCPA is non-cytotoxic, osteoconductive, and osseointegrative, and assists in the tissue repair. The HA/DCPA cement promoted bone formation and tissue filling of the entire defect area with degree of biomaterial degradation similar to that of TCP bioceramic, proving that HA/DCPA is equally suitable and successful for bone regeneration.

## Data Availability

All data sets were generated or analyzed in the current study.
